# Personality and socio-demographic variables in teacher burnout during the COVID-19 pandemic: a latent profile analysis

**DOI:** 10.1038/s41598-022-18581-2

**Published:** 2022-08-22

**Authors:** Camelia-Mădălina Răducu, Elena Stănculescu

**Affiliations:** Faculty of Psychology and Educational Sciences, University of Bucharest, 90 Panduri Street, 5th District, 030018 Bucharest, Romania

**Keywords:** Psychology, Health occupations

## Abstract

Although it is well-known that teaching is one of the most stressful jobs, teacher burnout during the COVID-19 pandemic has not been thoroughly investigated. The main aims of this study were to identify distinct teacher burnout profiles and examine their association with HEXACO personality factors and sociodemographic variables. Data were collected from 522 teachers (77% women; M_age_ = 37.45 years, *SD* = 9.28) in November 2021. Latent profile analysis (LPA) identified five latent profiles: *“No burnout risk”* (41.3%), *“Low burnout risk”* (21.9%), *“Cynics”* (7.7%), *“Exhausted and cynics”* (16.1%), and *“High burnout risk”* (13%). Our results showed a significant correlation between all six HEXACO personality traits and teacher burnout based on the variable-centered method, but the LPA highlighted that only the emotionality trait was antecedent of profile membership. In terms of sociodemographic variables, gender and rural/urban teaching environment did not have significant impact on teacher burnout profiles, but professional experience did. This study is the first to explore teacher burnout during the COVID-19 pandemic in relation to dispositional traits based on the HEXACO model using a person-centered approach. Our results can inform specialists about the role of emotionality in the occurrence of teacher burnout and the greater vulnerability of experienced teachers in the context of remote learning. Tailored programs of intervention are necessary.

## Introduction

The unprecedented situation generated by the coronavirus pandemic has led to a significant change in the quality of individuals’ professional and personal lives, bringing with it considerable dangers to their mental health^1^. Moreover, the prolonged situation of continuous evolution and the unpredictable changes generated by the spread of the coronavirus in its new forms are associated with family stress, loss of activities and occupations, social and economic instability, disturbance of the feeling of belonging, and new ways of working, all of which test people’s ability to recover and increase the risk of mental health difficulties^2–4^. However, for some professionals, these challenges have been even greater, with new working conditions adding extra stress to an already difficult situation^5^. These vulnerable groups have begun to take shape as studies on the effects of the pandemic on various social groups have progressed. In this regard, health care professionals, and social workers as well as teachers are considered to be the groups most exposed to symptoms of stress-related disorders^6^. Stress-related disorders are the main cause of burnout, a psychological syndrome emerging as a prolonged response to chronic interpersonal stressors on the job^7^. Moreover, the literature offers a solid evidence base supporting that burnout is more than just chronic job exhaustion but rather a depressive condition that requires special attention^8–10^.

### Teacher burnout and dispositional traits

Despite the fact that the teaching profession is already widely recognized as one of the most stressful professions and is highly prone to burnout and mental health disorders^11,12^ due to a high level of job stressors, the new conditions of online teaching, hybrid teaching, asynchronous classes, and social distancing classes have burdened them even more by putting them at risk of experiencing socio-contextual burnout^13^. This particular type of teacher burnout is characterized by three distinct symptoms— (i) exhaustion characterized by a lack of emotional energy and a feeling of being overwhelmed and tired at work, (ii) inadequacy in teacher–pupil interactions^14^—that affect teachers’ health and emotional well-being and (iii) cynicism represented by detachment from the job in general as well as from the teaching community^12^. The prevalence and specifics of teacher burnout need to be studied. For instance, despite the fact that reducing teacher burnout is a prominent topic of discussion among researchers, meta-analyses and systematic reviews have highlighted that the association between personality and teacher burnout has only been studied in the context of the Big Five framework^15,16^. The Big Five model is the dominant theory of personality^17^ and assumes that there are five domains that underlie one’s personality: openness (creative, curious, cultured), conscientiousness (organized, responsible), extraversion (sociable, assertive, energetic), agreeableness (kind, cooperative, trustworthy), and emotional stability (calm, confident, emotionless).

In terms of the relationship between teacher burnout and personality traits, emotional stability/neuroticism has been considered the strongest correlate for this construct^16,18^. More specifically, individuals with low emotional stability (high neuroticism) tend to experience negative and stressful emotions due their negative filter amplifying the impact of adverse events^18^, while individuals with high levels of emotional stability (low neuroticism) are protected from experiencing emotional exhaustion, depersonalization, and reduced personal accomplishment^19–21^. Concerning extraversion, meta-analyses conducted before the pandemic^22,23^ revealed that teachers with low levels of this dispositional trait tended to focus more on negative aspects of events and to use predominantly emotion-focused coping strategies. Furthermore, low levels of extraversion have been associated with reduced personal accomplishment^24,25^.

Teachers’ conscientiousness has been associated with two of the three domains of teacher burnout : depersonalization and reduced personal accomplishment^21,26^. In other words, teachers who have weaker willingness and who are less persistent about pursuing their goals are more likely to become cynical, withdraw from challenging situations to avoid stress and extra effort, and experience feelings of inadequacy and low job self-efficacy. The link between conscientiousness, job performance^27^, job satisfaction, work engagement, and teachers’ self-efficacy^28^ has shown that very conscientious teachers are less likely to feel low personal achievements regarding their work.

Agreeableness, an important dispositional trait in the teaching profession, provides teachers with the ability to build successful interpersonal relationships at work, be gentle, and be cooperative, behaviors that diminish the likelihood of experiencing detachment from the teaching community and inadequacy in teacher–pupil interactions^26,29^. In terms of openness to experience, the last area of the Big Five model, the literature has not established a clear link between this dispositional trait and teacher burnout. However, the increased intellectual curiosity and open-mindedness of the teachers with a high score for this domain predisposes them to seeing work challenges as opportunities rather than hindrances^30^. Moreover, the association of openness with emotion-focused coping strategies^31^ and implicitly with teacher burnout profiles^32^ requires a more detailed study of this connection.

Furthermore, as previous studies on teachers’ personalities have suggested, studies using newer and more extensive factorial models, such as the HEXACO model, would be preferable^15,33,34^. This six-dimensional model of human personality owes its uniqueness to its sixth dimension, namely the honesty/humility (H) scale. The other dimensions from the HEXACO model, which are similar to the factors in the Big Five model, are emotionality (E), extraversion (X), agreeableness (A), conscientiousness (C), and openness to experience (O).

In a comparison between the NEO Personality Inventory Revised (NEO PI-R; based on the Big Five model and the HEXACO model, it was suggested that although there is a substantial overlap in content between the two, they organize certain traits somewhat differently^35^. In this sense, HEXACO is more advantageous not because of the addition of the sixth H dimension but rather due to the composition of emotionality/neuroticism, which differs from the NEO PI-R model. In the HEXACO model, the E dimension includes more uniform and refined facets such as fearfulness, anxiety, dependence, and sentimentalism^33^. Concerning the H dimension, although it overlaps substantially with agreeableness from Big Five model, a meta-analysis of 400 studies showed that the H dimension provides important information about psychopathy, Machiavellianism, and narcissism^36^. As the personality constructs of the Dark Triad, these aspects have received little attention in studies on teachers’ personality domains. However, one study on the Dark Triad and burnout syndrome revealed that teachers with low levels of honesty/humility may be predisposed to antisocial acts and a lack of communication and empathy in the classroom environment, which makes them more likely to experience all three burnout symptoms^37^, but these assumptions have not been tested. In contrast, teachers with high scores for the H, C, and A dimensions and low scores for the E dimension are more likely to be empathetic, tolerant, altruistic, sincere, and organized teachers. These traits are important for the educational process in general but especially in stressful conditions such as those caused by the coronavirus pandemic. Unfortunately, there is a lack of investigations to shed more light on these connections, as one study only highlighted the mediating role of the H dimension between perceived organizational politics and job stress^38^, while another study found no link between the H dimension and emotional labor^39^.

In addition to the teachers' personality traits, their socio-demographic characteristics play a very important role in experiencing burnout symptoms. In this sense, some studies have concluded that female teachers are more vulnerable to stress and burnout^40^, as are teachers with less teaching experience in contrast to more experienced teachers^41^. However, some studies carried out at the beginning of the pandemic suggest that these reports were overturned by the pandemic context and that younger teachers, due to better digital skills, were less stressed in online teaching compairing to more experienced teachers^42^. In the same way, teachers from the urban environment were more advantaged in online teaching than those from the rural environment due to a better digital infrastructure, which prevented them from experiencing additional stress leading to burnout^43,44^. Furthermore, it was also suggested that the gender differences in the experience of burnout symptoms in the case of teachers were canceled by the context of online teaching during the pandemic^44,45^.

## The current study

As it has been established that teachers represent a group that is vulnerable to burnout during the COVID-19 pandemic^46^, the purpose of this study is to explore how personality traits explain clustering according to the continuum of no burnout to high burnout risk during the pandemic. The theoretical framework for our study is the job demands–resources model of burnout^47^. This model states that an imbalance between demands and resources—that is, high workload or work demands and low levels of physical (equipment), psychological (personality factors, work-related skills, job self-efficacy), social (support from managers and colleagues), and organizational (discretion related to tasks) resources—are associated with job strain.As mentioned above, previous studies conducted using the variable-centered approach showed correlations between teacher burnout and all dispositional traits assessed in the Big Five model but the relationship between the sixth factor included in HEXACO has not been specifically studied. In addition, few studies on teacher burnout using latent profile analysis (LPA) have been conducted. Therefore, the aims of the current study are to (i) verify the pattern of association among teacher burnout and personality traits using the variable-centered approach, (ii) explore how many profiles of teacher burnout we could find using the LPA approach, (iii) analyze whether personality traits depicted in the HEXACO model constitute antecedents of teacher burnout profiles, and (iv) investigate the association between teacher burnout profiles and socio-demographic variables (i.e., gender, professional experience, and teaching environment).

It is important to note that LPA has more advantages than the variable-centered method. Specifically, LPA is a mixture modelling technique that assumes that people can be typed with varying degrees of probability into categories that have different configural profiles of personal and/or environmental attributes, which in our study are all items of the Socio-Contextual Teacher Burnout Inventory (STBI). When the variable-centered method cannot identify how many categories are suitable, LPA presents an alternative approach that allows for the identification of relatively homogeneous sub-samples in a large population, permitting a more individualized identification of teachers in relation to burnout.

### Participants

Initially, 558 teachers were contacted and asked to be involved in this research, but 36 of them did not agree to participate. Therefore, the attrition rate was 6.5%. Our sample included 522 teachers (77% women; *M*_*age*_ = 37.45 years, *SD* = 9.28) who taught in kindergarten, primary, and middle schools. Their reported professional experience was less than one year (6.5%), between two and five years (13.4%), between five and 10 years (16.7%), between 10 and 20 years (25.7%), and more than 20 years (37.7%).

### Procedure

The whole study protocol was conducted in accordance with the Declaration of Helsinki, and approval for the study was granted by the corresponding author’s university ethics committee (no. 11/26.04.2021). The data were collected and processed, respecting all the rights and guarantees provided in EU Regulation 2016/679 and Organic Law 3/2018 of 5 December on the Protection of Personal Information and guarantee of digital rights. Data were collected in November 2021 using a convenience sampling method. The link to the online survey was posted with a short description of its purpose, the length of time needed to complete it, and invitations for others to share the link on the social networks of the targeted professional groups. All participants were voluntarily involved, with personal confidentiality guaranteed in all circumstances. After being informed of the research objectives and the anonymous nature of their answers, they gave their written informed consent prior to filling out the questionnaire. The teachers did not receive any remuneration for their participation and were informed that the research was for scientific purposes only.

### Measures

#### Teacher burnout

The STBI^14^ was used to measure teacher burnout. This nine-item scale (sample item: “With this work pace, I don’t think I’ll make it to the retiring age”) employed a Likert scale from 1 (completely disagree) to 7 (completely agree). The established three constructs were teacher exhaustion, cynicism toward the teacher community, and inadequacy in the pupil–teacher relationship. The Cronbach’s alpha for the entire scale was 0.93 (95%CI: 0.92, 0.94). The Cronbach’s alphas for each individual dimension were as follows: exhaustion (0.88; 95%CI: 0.87, 0.90), cynicism (0.77; 95%CI: 0.74, 0.80), and inadequacy (0.85; 95%CI: 0.83, 0.87). Previous studies^14,44^ have highlighted the construct validity of this scale In the current study, confirmatory factor analysis was performed using robust maximum likelihood estimation method. The results provided evidence for the good fitting to the data of the three-factor model proposed in the original study of the TSCBI validity^14^: CFI = 0.96, TLI = 0.94, RMSEA = 0.06, CI [0.05, 0.08], SRMSEA = 0.02, λs ranged between 0.54 and 0.87).

#### Teachers’ personality traits

The HEXACO-60 scale^34^ was employed to examine personality traits using six personality items: honesty-humility (H), emotionality (E), extraversion (X), agreeableness (A), conscientiousness (C), and openness to experience (O). Teachers were asked to mark their responses based on a 5-point Likert scale where 1 indicates strongly disagree and 5 indicates strongly agree. Good psychometric properties have been reported in the country in which the study took place (authors). For the present study, the subscale Cronbach’s alpha values were as follows: H (0.71; 95%CI: 0.68, 0.75), E (0.70; 95%CI: 0.65, 0.75), X (0.73; 95%CI: 0.68, 0.77), A (0.80; 95%CI: 0.75, 0.85), C (0.82; 95%CI: 0.75, 0.90), and O (0.76; 95%CI: 0.70, 0.81). In the current study, the construct validity of the six-factor model of HEXACO-60 was analyzed using the same technique as in previous studies^48–50^ namely principal axis factoring extraction method with varimax rotation. The results of the factorial analysis showed that the six common factors accounted for 35.2% of the item variance (similar to those reported for the original English version, i.e., 37.4% and 29.1%)^48^.

*Socio-demographic variables* such as gender, professional experience, and urban or rural teaching environment were also collected.

### Data analysis

Descriptive statistics, correlations, analyses of variance, robust tests of equality of means, and the Games–Howell post-hoc test were conducted using SPSS 28.0 (IBM Corp. Released 2021. IBM SPSS Statistics for Windows, Version 28.0. Armonk, NY: IBM Corp) The Games–Howell post-hoc test was applied to identify the differences among teacher burnout profiles in terms of mean profile indicators (i.e., all STBI items).. LPA was conducted to explore sets of mutually exclusive and exhaustive latent profiles using continuous indicator variables (i.e., all STBI items) using Mplus 8.7 software (MPLUS, Released 2021. Version 8.6. Los Angeles, CA: Muthén & Muthén). The robust maximum likelihood estimation method was used, as it produces robust standard errors to handle non-normally distributed data. We considered models with two to six classes, each with nine indicators, that is, all items of the STBI. To determine the number of teacher burnout profiles for optimal data analysis, we compared the goodness-of-fit indicators recommended by Nylund et al.^51^: log likelihood (LL), Akaike information criterion (AIC), Bayesian information criterion (BIC), sample size-adjusted BIC (SSA-BIC), and entropy (*R*^2^). We also performed supplementary tests—the adjusted Lo–Mendell–Rubin likelihood ratio test and a bootstrap likelihood ratio test—in order to compare the subsequent models. Additionally, solution stability was checked to assure the maximum likelihood solution could be replicated using multiple sets of random starting variables. Model identification was evaluated with 1000 sets of random starting values for all models, and 100 iterations and 100 solutions were retained for the final stage of optimization^52^. After identification of the profiles using multinomial logistic regression computed with the R3STEP procedure, we tested the predictive role of various types of teacher personality traits on profile membership. Baseline-category multinomial logistic regression indicates the increase in odds of membership in a target latent profile compared to other profiles for each one-unit increases in the predictor, that is, various types of teacher personality traits. Using the same procedure, we also examined the association between profile membership and socio-demographic variables, that is, gender, professional experience, and urban/rural teaching environment.

### Institutional review board statement

The study was conducted according to the guidelines of the Declaration of Helsinki and approved by the University of Bucharest Ethics Committee (no 11/26.04.2021).

### Informed consent statement

Informed consent was obtained from all subjects involved in the study.

## Results

### Descriptive statistics and correlation matrix

Descriptive statistics for the overall teacher burnout score and its dimensions as well as the correlation matrix are presented in Table 1. Correlation matrix highlighted that all personality traits were significantly related to teacher burnout (overall score) and its three dimensions. The prevalence in the five profiles according to the sociodemographic variables can be found in the Appendix. It should be noted that the prevalence of the high burnout risk profile was 13%. In a pre-pandemic study conducted with Finnish teachers, this prevalence was much lower (4%)^42^, while an early pandemic study conducted with Manitoban (Canadian) teachers showed that 17.7% of teachers had a high risk of developing burnout syndrome^45^. The current study conducted one year and a half after the onset of the pandemic, showed a higher prevalence than pre-pandemic, and less prevalence than early pandemic. The higher prevalence is not surprising given the magnitude of job demands in terms of remote learning conditions and the uncertainties and insecurities generated by this unpredictable and challenging situation. At the same time, the less prevalence obtained in the current study comparing to early pandemic Manitoban study can also be explained by the cultural differences.

**Table 1:** Correlation matrix between research variables.

Variables	1	2	3	4	5	6	7	8	9	10
Exhaustion	–									
Cynicism	0.79**	–								
Inadequacy	0.80**	0.72**	–							
Total burnout	0.94**	0.90**	0.91**	–						
H	– 0.33**	– 0.33**	– 0.41**	– 0.39**	–					
E	0.30**	0.17**	0.23**	0.26**	0.03	–				
X	− 0.45**	− 0.39**	− 0.44**	− 0.46**	0.18**	− 0.24**	–			
A	− 0.44**	− 0.41**	− 0.43**	− 0.46**	0.35**	− 0.26**	0.35**	–		
C	− 0.12**	− 0.13*	− 0.21**	− 0.16**	0.38**	0.08	0.12**	− 0.02	–	
O	− 0.26**	− 0.27**	− 0.33**	− 0.31**	0.30**	− 0.01	0.46**	0.30**	0.36**	–

**p < 0.01.

### Latent profile solutions

Fit indices of two- to five-profile solutions of LPA are shown in Table 2. Progressive improvement of LL, AIC, SSA-BIC, and entropy was observed up to the six-profile solution, but the best loglikelihood value was replicated for only the first five profiles. Consequently, these results lent support for the five-profile solution as the best fitting model for the present study’s data. Additionally, in the five-profile model, the average latent profile probabilities for the most likely profile were 0.98, 0.95, 0.92, 0.89, and 0.92. All were well-above the cutoff (> 0.80) recommended by Watson et al.^53^.

**Table 2:** Model fit information for latent profile analysis.

No of profiles	Free parameters	LL replicated	LL	AIC	BIC	SSA-BIC	*R*^*2*^	BLRT	*p*
2	28	Yes	−8248.89	16,553.78	16,672.99	16,584.11	0.936	−9549.79	0.00
3	38	Yes	−7886.47	15,848.95	16,010.74	15,890.12	0.940	−8248.89	0.00
4	48	Yes	−7788.97	15,673.94	15,878.31	15,725.95	0.920	−7886.47	0.00
**5**	**58**	**Yes**	**−7718.38**	**15,552.76**	**15,799.70**	**15,615.60**	0**.923**	**−7788.97**	0**.00**
6	68	No	−7657.35	−7657.35	15,740.23	15,524.38	0.894	−7705.58	0.00

Information for the best fitting model is in bold.

### Five-profile model of teacher burnout risk

**Figure 1:**
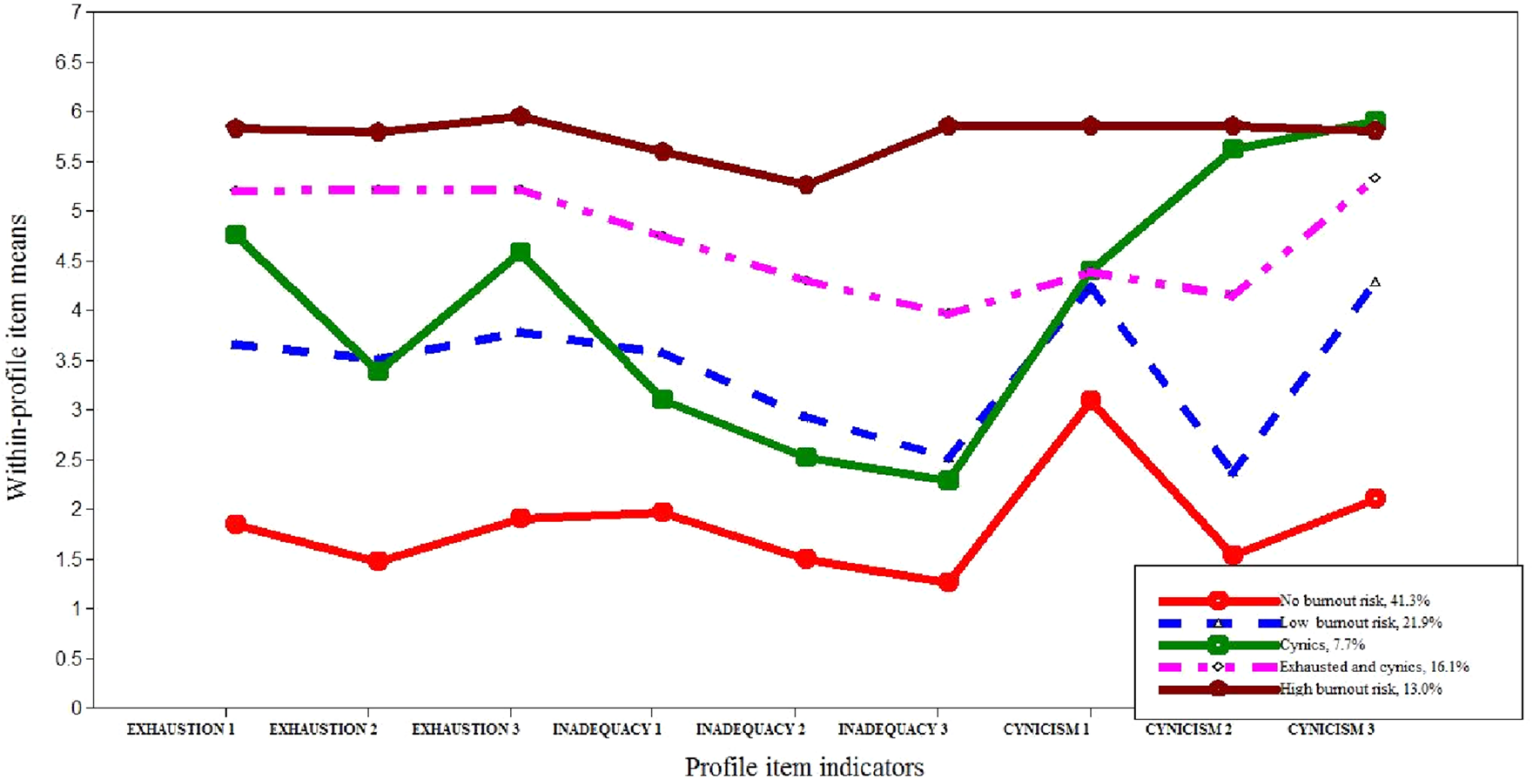
Parameter estimates for the five-profile model of teacher burnout risk and within-profile item means.

The five identified latent profiles of teacher burnout risk are shown in Fig. 1. Indicators of profile membership were all items of each subscale of STBI.

Parameter estimates for overall item respectively within-profile item means for the five-profile model are shown in Table 3. The first profile, *“No burnout risk”*, included 41.3% of the teachers and was characterized by very low levels of each item (scores < 2), except for the first item of the cynicism subscale (“I’m disappointed in our teacher community’s ways of handling our shared affairs.”), which had a low score. The second profile, *“Low burnout risk”*, included 21.9% of the teachers and was defined by low scores for all items except the first and third items of the cynicism subscale (“I’m disappointed in our teacher community’s ways of handling our shared affairs.”, “In spite of several efforts to develop the working habits of our teacher community they haven’t really changed.”), which had moderate scores. All other items had scores less than 2. The third profile, *“Cynics”*, included 7.7% of the teachers. In terms of profile indicators, moderate scores were obtained for exhaustion, low scores for inadequacy, and moderate to high-moderate for cynicism. Notably, the score for the third cynicism item was the highest for all profiles (score around 6).

**Table 3:** Parameter estimates for the five-profile model of teacher burnout risk.

Profile prevalence		First profile	Second profile	Third profile	Fourth profile	Fifth profile
		n = 216	n = 114	n = 40	n = 84	n = 68
Profile indicators	Overall item, means (*SD*)	Within-profile means estimate (*SE*)				
Exhaustion 1	3.51 (1.89)	1.83 (0.06)	3.66 (0.17)	4.74 (0.25)	5.19 (0.12)	5.82 (0.21)
Exhaustion 2	3.22 (1.99)	1.47 (0.04)	3.50 (0.23)	3.38 (0.33)	5.20 (0.24)	5.78 (0.16)
Exhaustion 3	3.57 (1.84)	1.89 (0.07)	3.77 (0.20)	4.58 (0.19)	5.21 (0.09)	5.95 (0.17)
Inadequacy 1	3.31 (1.73)	1.95 (0.09)	3.56 (0.12)	3.09 (0.31)	4.73 (0.23)	5.59 (0.13)
Inadequacy 2	2.83 (1.79)	1.49 (0.07)	2.92 (0.19)	2.52 (0.37)	4.29 (0.22)	5.26 (0.17)
Inadequacy 3	2.64 (1.82)	1.26 (0.03)	2.51 (0.18)	2.27 (0.22)	3.96 (0.26)	5.85 (0.33)
Cynicism 1	4.00 (1.77)	3.09 (0.15)	4.23 (0.12)	4.39 (0.28)	4.37 (0.26)	5.84 (0.16)
Cynicism 2	3.01 (1.89)	1.53 (0.05)	2.36 (0.13)	5.61 (0.19)	4.14 (0.31)	5.86 (0.13)
Cynicism 3	3.86 (1.90)	2.10 (0.15)	4.27 (0.16)	5.90 (0.22)	5.32 (0.16)	5.80 (0.11)

First profile: “No burnout risk”; Second profile: “Low burnout risk”; Third profile: “Cynics”; Fourth profile: “Exhausted and cynics”; Fifth profile: “High burnout risk”.

The fourth profile, *“Exhausted and cynics”*, included 16.1% of teachers. The defining features were higher levels of exhaustion compared to previous profiles. Specifically, all items of this subscale had a score greater than 5 and high-moderate to high scores for cynicism. The final profile, *“High burnout risk”*, included 13% of the teachers and was characterized by higher levels of all indicators compared to the previous profiles. More precisely, all items registered scores greater than 5.

Supplementary analyses were performed to determine the differences among means of each indicator of profile membership (i.e., robust tests of equality of means showed that all Welch scores were significant). More specifically, significant differences among profiles in terms of each indicator were found (Welch values ranged from 51.67 to 660.02, *p* < 0.001). Furthermore, pairwise comparison tests showed significant differences between almost all pairs of means with a few exceptions. In the case of Profiles 2 (Low burnout risk) and 3 (Cynics), the Games–Howell test revealed no significant differences in the case of the second exhaustion item, first cynicism item, and all items on the inadequacy subscale. The same pattern was found between Profiles 3 (Cynics) and 5 (High burnout risk) for the second and third items of cynicism (“I often feel like an outsider in my work community.” and “In spite of several efforts to develop the working habits of our teacher community they haven’t really changed.”). It was also observed that in the case of first item for cynicism (“I’m disappointed in our teacher community’s ways of handling our shared affairs.”), similar scores indicating a high-moderate level were obtained in the case of Profiles 2 (Low burnout risk), 3 (Cynics), and 4 (Exhausted and cynics).

### Antecedents of latent profiles

Using the no burnout risk profile as a reference, we found that only emotionality was an antecedent of teacher burnout risk profile membership. As shown in Table 4, a significant increase in the contribution of the emotional factor on all other profiles was obtained (OR > 1, *p* < 0.05). In other words, emotionality had a more pronounced impact on the Low burnout risk, Cynics, Exhausted and cynics, and High burnout risk profiles than No burnout risk profile. Our results highlighted odds ratios greater than 1, but nonsignificant for: (i) the H dimension in the case of Profile 2 (Low burnout risk), (ii) consciousness in the case of Profile 3 (Cynics), and (iii) openness in the case of the Profile 5 (High burnout risk). Based on the *p* value mentioned above, the personality traits of H, C, and O did not have a predictive role in profile membership. The remaining personality traits—extroversion, agreeability, and openness—did not have a significantly higher contribution to any profile membership (OR < 1, *p* > 0.05). Although robust tests of equality of means have shown that there are statistically significant differences between all six dispositional traits among the five profiles, only the emotionality trait was an antecedent of the profile membership.

**Table 4:** Effects of predictors on membership in latent profiles of teacher burnout risk.

Burnout profile	Odds ratio (OR)	LL2.5%	UL2.5%
**Reference profile: low burnout risk**			
**Low burnout risk**			
Extraversion	0.92	0.87	0.98
Emotionality	1.04***	1.02	1.09
Consciousness	0.87	0.82	0.93
Agreeability	0.91	0.86	0.97
Openness	0.95	0.88	1.02
Humility	1.03***	1.01	1.10
**Moderate burnout risk**			
Extraversion	0.88	0.82	0.94
Emotionality	1.12***	1.03	1.23
Consciousness	1.06***	1.04	1.16
Agreeability	0.90	0.84	0.96
Openness	0.91	0.85	0.97
Humility	0.92	0.87	0.98
**High burnout risk**			
Extraversion	0.82	0.77	0.87
Emotionality	1.22***	1.12	1.32
Consciousness	0.97	0.91	1.03
Agreeability	0.88	0.77	0.87
Openness	0.99	0.92	1.07
Humility	0.86	0.82	0.91

Odds ratios (OR), 95% confidence interval for the effects of the six personality factors on teacher burnout profile membership.

*LL *lower limit of the confidence interval, *UL *upper limit.

**p < 0.01; ***p < 0.001.

### Sociodemographic variables as antecedents of teacher burnout profile membership

Professional experience had an increasing contribution to each profile. More precisely, teachers with the highest level of experience had higher odds than less experienced teachers (< 2 years) of belonging to the Low burnout risk (OR = 1.35; 95%CI: 1.10, 1.67), Cynics (OR = 1.53; 95%CI: 1.12, 2.11), Exhausted and cynics (OR = 1.85; 95%CI: 1.37, 2.50), and High burnout risk (OR = 1.98; 95%CI: 1.56, 2.73) profiles than to the no burnout risk profile. In addition, our findings revealed that professional experience was the only sociodemographic variable that had a significant impact on profile membership.

Although ORs obtained for gender and teaching environment were greater than 1, they were not significant (*p* < 0.05). Consequently, gender and teaching environment were not antecedents of teacher burnout profiles. This is interesting because we could not confirm the well-known gender differences in burnout^31^. Moreover, men even had a higher prevalence than women (as shown in the Appendix) for all profiles except the first one (no burnout risk), but the differences were not significant. Basically, our findings proved that gender differences in teacher burnout have disappeared in the context of the COVID-19 pandemic.

## Discussion

Our results revealed five latent profiles of teacher burnout for in-service teacher burnout during the COVID-19 pandemic. Five latent burnout profiles were also identified in previous studies among Finnish^42^ and Manitoban/Canadian/^45^ teachers. In addition, our findings revealed that 13% of the teachers in our sample suffered from an increased risk of developing burnout syndrome during the COVID-19 pandemic. These results are not surprising since other studies have highlighted the significant risk of experiencing work-related stress among education professional in the pre-pandemic^12,21,32^ and early pandemic^45,54^ periods.

The most protected teachers were those from the first profile, namely, no burnout risk. Being the most widespread profile among teachers, with a sample share of 41.3%, we can conclude that most teachers found internal resources to adapt to teaching in crisis situations. They showed significantly lower levels of exhaustion, inadequacy in the teacher–student relationship, and cynicism toward the professional community than those in the other profiles. Interestingly, Pyhalto’s^42^ study conducted before the pandemic revealed that 47% of teachers were not at risk of developing burnout syndrome, while Sokal’s^45^ study conducted in the first months of the pandemic found that only about 10% of teachers were still characterized by low burnout symptoms. Thus, our study conducted approximately one year into the pandemic reveals a prevalence of a low risk of burnout similar to before the pandemic^32^. Thus, we can determine that over time, teachers have found resources to protect themselves and adapt to new teaching conditions. Another explanation could be related to the different cultures, the present study was conducted in a different culture from the Finnish and the Canadian, respectively Canadian, so the terms of comparability are quite difficult to apply. However, our study did not aim at a cross-cultural analysis but at identifying latent burnout profiles according to general personality traits.

The second profile, Low burnout risk, included 21.9% of teachers. Analyzing the responses to the items for this profile, most of the burnout symptoms were fueled by frustration with how the professional community has managed the pandemic crisis. Similar to the previous profile, before the pandemic, slightly more teachers were included in this profile (25%)^42^, while early in the pandemic, only 17.2% of teachers had a low burnout risk^54^.

The third profile, Cynics, included 7.7% of the teachers. They were characterized by low inadequacy, moderate exhaustion, and moderate to high-moderate cynicism. There is no exact match for this profile in previous studies^42,45^, so we can assume that the pandemic has led to a new profile of teachers in terms of burnout symptoms. Given that the score for the third item of cynicism (“In spite of several efforts to develop the working habits of our teacher community they haven’t really changed.”) was the highest of all profiles, there is a marked loss of hope among teachers related to work habits in the professional community resulting from the new conditions imposed by the COVID-19 pandemic.

The fourth profile, Exhausted and cynics (16.1%), included teachers with higher levels of exhaustion and cynicism compared to previous profiles combined with moderate inadequacy in teacher–pupil interactions. Comparing this profile with the burnout profiles from previous studies, before the pandemic, only 6% of Finnish teachers reported high levels of exhaustion and cynicism^42^. In the first few months of the pandemic, 28% of Canadian teachers declared themselves intensely affected by these symptoms^45^. In our study, we observed that 16.1% of the teachers were exhausted and cynical toward the professional community after the first year of teaching during the pandemic.

The fifth profile*,* high burnout risk, included 13% of the teachers and was characterized by higher levels of all indicators compared to the previous profiles. Our percent of burned out teachers was much higher than the pre-pandemic values found in the Finnish study, which reported that only 4% teachers fit this profile^42^, but lower than the early pandemic Manitoban/Canadian study, which reported that an alarming 17.7% of teachers had high burnout risk^45^. Additionally, the third item of this profile (“I often feel I have failed in my work with pupils”), the inadequacy scale, had the highest value, with teachers reporting an increased feeling of failure in their relations with pupils. The same pattern was obtained for the third item of the exhaustion scale, with teachers describing high symptoms of somatic stress. Taking into account the JD-R model^47^, these changes are likely related to the institution of remote learning during the COVID-19 pandemic, specifically the pressure to adapt teaching methods to the use of digital content. These teachers experienced limited resources, a lack of administrative support, and a lack of preparedness to build virtual relations with pupils. Consequently, they had a higher risk of developing burnout, which led to somatic symptoms of stress in the form of physical tension, restlessness, nervousness, and sleep problems.

The data were collected in the context of the pandemic, when remote learning conditions, a state of confusion and fear regarding COVID-19, and the dramatic effects of the disease were already widespread. Thus, it is not surprising that 13% of our study sample was included in the high risk of burnout profile. Previous studies conducted before the pandemic observed much lower percentages of teachers experiencing a high risk of burnout^32,55^. Thus, teaching in pandemic conditions greatly increased the risk of teachers developing symptoms of burnout and depression^9,10^, but further research is needed. This finding is also supported by Weißenfels et al.’s^56^ study on German teachers, which measured teachers’ burnout symptoms at two different times, before and during the pandemic, and revealed that the burnout components of depersonalization and lack of accomplishment significantly increased from the pre- to post-COVID-19 outbreak, but emotional exhaustion did not.

The present study has the advantage of providing a more refined perspective on teacher burnout profiles by involving each STBI item in the profiles, whereas previous studies only used the subscale overall scores^42,45^. Thus, we were able to see which of the items specific to each subscale had the highest scores in each profile. Browsing through the profiles, we can see that in the first four profiles, the first item in the inadequacy scale (“Dealing with problem situations considering my pupils often upsets me”) had higher scores than the other two items of the subscale. Because the other two items on the inadequacy scale refer specifically to a lack of skill as a teacher and failure in relationships with students, it seems that the pandemic has exacerbated problematic situations regarding the relationship with students. This plausible explanation must be explored in future studies. One of the most reported challenges in teacher–student interaction was maintaining attention and motivation in remote teaching^57^. Furthermore, in the cynics and exhausted and cynics profiles, the third item from the cynicism subscale (“In spite of several efforts to develop the working habits of our teacher community they haven’t really changed.”) recorded higher scores than the other two items of the subscale. Moreover, even in the low burnout risk profile, the third item on the cynicism scale had a score greater than 4. As mentioned before, in the fifth profile, all the items of the cynicism subscale had higher scores, ranging from 5.80 to 5.86. Thus, our findings provide support for the idea that teachers felt burned out because of their disappointment with the teaching community’s methods of handling shared affairs and because the working habits of the teaching community have not really changed. Although cynicism was much lower in previous studies^9,21,42,55^, in our research group, the scores were very high. One explanation could be that the pandemic period surprised the education administration, and teachers were disappointed with how they chose to implement new teaching strategies and the resources they made available to teachers.

Furthermore, while previous studies aimed at clustering teacher burnout with coping strategies^42^ and job demands versus resources^54^, our study aimed to verify the pattern of association between teacher burnout and personality traits using both the variable-centered approach and person-centered approach. The last one was applied to verify which personality traits described in the HEXACO model are antecedents of teacher burnout profiles. In this regard, our findings highlighted significant correlations between personality traits and teacher burnout as well as significant differences among profiles depending on the six personality traits. In the case of the first five dimensions (which are similar to the Big Five Model), previous studies have established the connection between these traits and teacher burnout^16,18,19,29^. In the case of the H dimension, this is the first study to highlight this dimension’s correlation to teacher burnout.

Moreover, although there were significant differences among the profiles in terms of personality traits, only one, emotionality, has been shown to have a predictive role in teacher burnout clustering. As presented in the HEXACO model^33^, the emotionality trait describes the fear of physical dangers and experience of anxiety in response to life stressors, both of which are characteristic of the pandemic crisis. Added to these is the need for emotional support or to be detached. Thus, unlike the neuroticism factor in the Big Five Model, which focuses on anger, impulsivity, and vulnerability^25^, the HEXACO model is better at capturing workplace behavior^58^. Thus, high scores on the emotionality subscale predispose teachers to experiencing burnout. These results are in line with previous studies that have established the increased negative emotionality of teachers as an important predictor of developing burnout syndrome^18–21,37^. In other words, teachers with increased negative emotionality indicated that anxiety, dependence, low courage, and sentimentalism made them experience more fear of physical danger, more anxiety in response to life stressors, and more emotionally dependence on other factors that significantly contribute to the development of symptoms of emotional exhaustion, cynicism, and inadequacy in their relationships with their students. Thus, our findings support the results of pre-pandemic studies indicating that emotional stability was the strongest correlate of teacher burnout^16,18,29,59,60^. In other words, teachers with low emotional stability have tended to experience more negative and stressful emotions while teaching during the pandemic, which has made them far more vulnerable to emotional exhaustion, cynicism, reduced personal accomplishment, and inadequacy^20,21^.

Another finding of our research is that extraversion did not have a statistically significant impact on teacher burnout profile membership, although previous studies based on the variable-centered approach^21,24^ and meta-analyses^16,23^ have shown that there is a negative association between teacher burnout and extraversion. Thus, we can conclude that during the pandemic, extraversion did not significantly prevent teachers from developing symptoms of burnout. One explanation for this finding could be that in the HEXACO model, individuals with increased extraversion are described as people who enjoy social gatherings and physical interactions^33^. However, social interactions were intensely limited in the context of online teaching during the pandemic.

Similarly, the A and C dimensions were negatively correlated with teacher burnout , as shown in previous studies^16,21,30,59,61–63^, but they did not make a significant contribution to profile membership. This aspect needs to be studied in future longitudinal research to see how the relationship between these personality traits and teacher burnout evolves over time. However, a plausible explanation for the results obtained could be that the rather high prevalence of teachers with high scores for the cynicism dimension of burnout indicates an acute perception of a lack of resources, and the management of the imbalance between high job demands and less resources^54^ was not influenced by agreeableness and conscientiousness in the present study. In addition, in the fifth profile, all teacher burnout sub-dimensions had high scores. In other words, conscientiousness and agreeableness did not help teachers feel less exhausted, inadequate, or uncynical.

Concerning socio-demographic variables, despite the fact that previous studies have shown that female teachers are more likely to experience higher levels of work stress and exhaustion than male teachers^40^, our findings revealed that that the gender differences in the experience of stress and burnout seem to have been absorbed by the crisis context^56^, as was also suggested by the meta-analysis conducted by Ozamiz-Etxebarria et al.^64^. Furthermore, concerning the professional experience variable, pre-pandemic studies found that greater professional experience was positively associated with lower burnout risk^32,65^. However, it seems that in this study, older (more experienced) teachers might have experienced more difficulties in adapting to e-learning systems than younger teachers with stronger digital skills^66,67^, increasing their risk of developing burnout symptoms. Another explanation could be that before the pandemic, the main stressor for younger teachers was student misbehavior in the classroom^21,68^. Student misbehavior may be easier to manage in remote teaching, but further research is needed to verify this assumption. Furthermore, concerning the teaching environment, although some studies from the beginning of the pandemic suggested that rural teachers were disadvantaged and prone to stress due to a lack of infrastructure for broadband access and technological equipment^43^, it seems that in our study, teachers from these environments found in a similar manner to those teaching in the urban schools.

As can be seen, there are many interconnected aspects between teacher burnout and personality traits. While personality assessments have been used in the personnel selection process of the organizational environment for several decades, they are absent in the educational environment^64^. This could also be the reason why studies on teacher personality and its connection with different outcomes are so rare. Most of the studies in the educational field have focused on the inverse relationship between emotional intelligence and teachers’ burnout^69^. In a previous study, we even identified positive emotionality, well-being and self-control, basic traits of emotional intelligence, as the main protective factors against experiencing burnout^70^. However, the experience of burnout symptoms also depends on other internal factors besides teachers’ emotional intelligence^26^. In this regard, our study contributes to the development of the literature on both teacher burnout, a topic of great interest during periods of struggle such as the coronavirus pandemic, and on the literature on teacher personality, which is still controversial. Furthermore, our study encompasses various aspect that are new to the field. Firstly, to our knowledge, the present study is the first to investigate the link between teacher burnout and the HEXACO dimensions. Secondly, using a person-centered approach, our findings expand the previous research on teacher burnout by showing more refined contextualized differences by specifically analyzing the STBI items that contributed to teacher burnout profiles. Thirdly, the variable-centered approach revealed differences between profiles for all six HEXACO dimensions. To our knowledge, this is the first study to investigate the association between the H dimension and teacher burnout. In this regard, we found that the H dimension is associated with the risk of developing burnout symptoms. Fourthly, in line with previous studies^16,22,23,59^, all personality traits were correlated with teacher burnout (using the variable-centered approach). However, in terms of the antecedents of teacher burnout profiles (the person-centered approach), our results revealed that only emotionality had a significant contribution to profile membership*.* Fifthly, our study reveals that male and female teachers were equally affected with burnout symptoms during pandeming, with males even experiencing slightly more symptoms, but this difference was not statistically significant. This finding confirms the results of a meta-analysis from the beginning of the pandemic, which showed that both male and female teachers experienced similar burnout symptoms^64^.

Concerning the practical implications of this study, there is disagreement as to whether the practices in the field of organizational psychology can be successfully applied in educational systems and institutions given the unique and vocational nature of the teaching process^16,29^. However, our study joins the few previous studies^12,18,21,28,29,55^ that have claimed that districts and schools should take personality trait measures, along with other valid indicators, into consideration for improving the teacher hiring process. To our knowledge, this is the first study to profile the risk of developing burnout among teachers during the coronavirus pandemic based on personality traits. Thus, with the alarming burdening of the teaching profession^71,72^, the need to develop new and efficient continuous professional development programs has also increased, especially considering that the effectiveness of pre-pandemic interventions to reduce teacher burnout was minimal^73^. These interventions should focus on the development of emotional self-regulation, managing anxiety and emotional dependence, and healthy emotional bonds with others, and thus increasing stable emotionality.

The current study is not devoid of limitations. First, our sample is not representative of the entire population, and thus the findings cannot be generalized. Second, the cross-sectional design precludes identifying causal relationships between variables. Thus, a longitudinal study is necessary to examine how the relationship between teachers’ personality traits and teacher burnout evolve after the period of remote learning and the coronavirus pandemic end. In addition, future studies based on clinical interviews should use other measures of teacher burnout besides self-reports.

## Conclusions

In summary, our study expands the empirical body of research on teacher burnout risk^12,32,45,55,74^ by being the first to explore teacher burnout in relation to personality traits based on the HEXACO model during the COVID-19 pandemic. Our results showed that (i) 13% of the teachers in our sample presented a high risk of burnout, (ii) emotionality and professional experience were predictors of teacher burnout profile, (iii) and burnout was independent of teacher gender. In other words, during the challenging conditions of teaching during the pandemic, male teachers were as equally affected by burnout symptoms as female teachers. Moreover, given that the pandemic is affecting the entire word simultaneously, this study calls for short- and long-term interventions for vulnerable groups to work stress such as teachers.

## Supplementary Information

Supplementary Information.

## Data Availability

The data are available for those who want to see it with justified reasons. Kindly contact the corresponding author.

## References

[1] Xiong J (2020). Impact of COVID-19 pandemic on mental health in the general population: A systematic review. J. Affect. Disord..

[2] Pfefferbaum B, North CS (2020). Mental health and the Covid-19 pandemic. N. Engl. J. Med..

[3] Stănculescu E (2021). Fear of COVID-19 in Romania: Validation of the Romanian version of the fear of COVID-19 scale using graded response model analysis. Int. J. Ment. Heal. Addict..

[4] Vujčić I (2021). Coronavirus disease 2019 (COVID-19) epidemic and mental health status in the general adult population of Serbia: A cross-sectional study. Int. J. Environ. Res. Public Health.

[5] Chen H (2020). Are you tired of working amid the pandemic? The role of professional identity and job satisfaction against job burnout. Int. J. Environ. Res. Public Health.

[6] Du J (2020). Mental health burden in different professions during the final stage of the COVID-19 lockdown in China: Cross-sectional survey study. J. Med. Internet Res..

[7] Maslach C, Leiter MP (2016). Understanding the burnout experience: Recent research and its implications for psychiatry. World Psychiatry.

[8] Bianchi, R., Schonfeld, I. S. & Laurent, E. Burnout syndrome and depression. in *Understanding Depression*. 187–202 (Springer, 2018).

[9] Bianchi R (2021). Is burnout a depressive condition? A 14-sample meta-analytic and bifactor analytic study. Clin. Psychol. Sci..

[10] Schonfeld IS, Bianchi R (2016). Burnout and depression: Two entities or one?. J. Clin. Psychol..

[11] Ghanizadeh A, Jahedizadeh S (2016). EFL teachers’ teaching style, creativity, and burnout: A path analysis approach. Cogent. Educ..

[12] Schonfeld, I. S., Bianchi, R. & Luehring-Jones, P. Consequences of job stress for the mental health of teachers. in *Educator Stress*. 55–75 (Springer, 2017).

[13] Pietarinen J, Pyhältö K, Haverinen K, Leskinen E, Soini T (2021). Is individual- and school-level teacher burnout reduced by proactive strategies?. Int. J. School Educ. Psychol..

[14] Pietarinen J, Pyhältö K, Soini T, Salmela-Aro K (2013). Reducing teacher burnout: A socio-contextual approach. Teach. Teach. Educ..

[15] Göncz L (2017). Teacher personality: A review of psychological research and guidelines for a more comprehensive theory in educational psychology. Open Rev. Educ. Res..

[16] Kim LE, Jörg V, Klassen RM (2019). A meta-analysis of the effects of teacher personality on teacher effectiveness and burnout. Educ. Psychol. Rev..

[17] John OP, Donahue EM, Kentle RL (1991). Big five inventory. J. Pers. Soc. Psychol..

[18] Bianchi R (2018). Burnout is more strongly linked to neuroticism than to work-contextualized factors. Psychiatry Res..

[19] Bianchi R, Manzano-García G, Rolland J-P (2021). Is burnout primarily linked to work-situated factors? A relative weight analytic study. Front. Psychol..

[20] Jin YY, Noh H, Shin H, Lee SM (2015). A typology of burnout among Korean teachers. Asia-Pac. Edu. Res..

[21] Kokkinos CM (2007). Job stressors, personality and burnout in primary school teachers. Br. J. Educ. Psychol..

[22] Connor-Smith JK, Flachsbart C (2007). Relations between personality and coping: A meta-analysis. J. Pers. Soc. Psychol..

[23] García-Carmona M, Marín MD, Aguayo R (2019). Burnout syndrome in secondary school teachers: A systematic review and meta-analysis. Soc. Psychol. Educ..

[24] Foley C, Murphy M (2015). Burnout in Irish teachers: Investigating the role of individual differences, work environment and coping factors. Teach. Teach. Educ..

[25] RuggeroAndrisano R, Crescenzo P, Iervolino A, Mossi P, Boccia G (2018). Predictability of big five traits in high school teacher burnout detailed study through the disillusionment dimension. Epidemiol. Biostat. Public Health.

[26] Pishghadam R, Sahebjam S (2012). Personality and emotional intelligence in teacher burnout. Span. J. Psychol..

[27] Judge TA, Rodell JB, Klinger RL, Simon LS, Crawford ER (2013). Hierarchical representations of the five-factor model of personality in predicting job performance: Integrating three organizing frameworks with two theoretical perspectives. J. Appl. Psychol..

[28] Perera HN, Granziera H, McIlveen P (2018). Profiles of teacher personality and relations with teacher self-efficacy, work engagement, and job satisfaction. Pers. Individ. Differ..

[29] Cano-García FJ, Padilla-Muñoz EM, Carrasco-Ortiz MÁ (2005). Personality and contextual variables in teacher burnout. Pers. Individ. Differ..

[30] Zimmerman RD (2008). Understanding the impact of personality traits on individuals’ turnover decisions: A meta-analytic path model. Pers. Psychol..

[31] Antoniou, A.-S., Antoniou, A.-S. G. & Cooper, C. L. *Coping, Personality and the Workplace: Responding to Psychological Crisis and Critical Events*. Vol. 5. 345–367 (2017).

[32] Pyhältö K, Pietarinen J, Haverinen K, Tikkanen L, Soini T (2020). Teacher burnout profiles and proactive strategies. Eur. J. Psychol. Educ..

[33] Ashton MC, Lee K (2007). Empirical, theoretical, and practical advantages of the HEXACO model of personality structure. Pers. Soc. Psychol. Rev..

[34] Lee, K., Ashton, M. C., & Wilfrid Laurier University Press. *The H Factor of Personality: Why Some People are Manipulative, Self-Entitled, Materialistic, and Exploitive—and Why It Matters for Everyone*. (Wilfrid Laurier University Press, 2013).

[35] Gaughan ET, Miller JD, Lynam DR (2012). Examining the utility of general models of personality in the study of psychopathy: A comparison of the HEXACO-PI-R and NEO PI-R. J. Pers. Disord..

[36] Howard MC, Van Zandt EC (2020). The discriminant validity of honesty-humility: A meta-analysis of the HEXACO, Big Five, and Dark Triad. J. Res. Pers..

[37] Čopková R (2021). Burnout syndrome and dark triad at schools: Engineers as teachers of vocational technical subjects. ERIES J..

[38] Wiltshire J, Bourdage JS, Lee K (2014). Honesty-humility and perceptions of organizational politics in predicting workplace outcomes. J. Bus. Psychol..

[39] Sohn H-K, Lee TJ (2012). Relationship between HEXACO personality factors and emotional labour of service providers in the tourism industry. Tour. Manag..

[40] Klassen RM, Chiu MM (2010). Effects on teachers’ self-efficacy and job satisfaction: Teacher gender, years of experience, and job stress. J. Educ. Psychol..

[41] Capri B, Guler M (2018). Evaluation of burnout levels in teachers regarding socio-demographic variables, job satisfaction and general self-efficacy. Eurasian J. Educ. Res..

[42] Pyhältö K, Pietarinen J, Haverinen K, Tikkanen L, Soini T (2020). Teacher burnout profiles and proactive strategies. Eur. J. Psychol. Educ..

[43] Hayes SD, Flowers J, Williams SM (2021). “Constant communication”: Rural principals’ leadership practices during a global pandemic. Frontiers.

[44] Rǎducu C-M, Stǎnculescu E (2022). Teachers’ burnout risk during the COVID-19 pandemic: Relationships with socio-contextual stress—A latent profile analysis. Front. Psych..

[45] Sokal L, Babb J, EblieTrudel L (2021). Latent profile analysis of Manitoban teachers’ burnout during the COVID-19 pandemic. Front. Psychiatry.

[46] Beames JR, Christensen H, Werner-Seidler A (2021). School teachers: The forgotten frontline workers of Covid-19. Australas. Psychiatry.

[47] Demerouti E, Bakker AB, Nachreiner F, Schaufeli WB (2001). The job demands-resources model of burnout. J. Appl. Psychol..

[48] Ashton M, Lee K (2009). The HEXACO-60: A short measure of the major dimensions of personality. J. Pers. Assess..

[49] Drammen Psychiatric Centre, Drammen, Norway & Ørnfjord, M. The Norwegian HEXACO-PI-R: Psychometric properties and relationships with the Big Five Inventory. *Scand. Psychol.* **5**, 356–378 (2018).

[50] Skimina E, Strus W, Cieciuch J, Szarota P, Izdebski P (2020). Psychometric properties of the Polish versions of the HEXACO-60 and the HEXACO-100 personality inventories. Curr. Issues Pers. Psychol..

[51] Nylund KL, Asparouhov T, Muthén BO (2007). Deciding on the number of classes in latent class analysis and growth mixture modeling: A Monte Carlo simulation study. Struct. Equ. Model..

[52] Morin AJ, Arens AK, Marsh HW (2016). A bifactor exploratory structural equation modeling framework for the identification of distinct sources of construct-relevant psychometric multidimensionality. Struct. Equ. Model..

[53] Watson D (1995). Testing a tripartite model: II. Exploring the symptom structure of anxiety and depression in student, adult, and patient samples. J. Abnormal Psychol..

[54] Sokal LJ, Trudel LGE, Babb JC (2020). Supporting teachers in times of change: The job demands-resources model and teacher burnout during the COVID-19 pandemic. Int. J. Contemp. Educ..

[55] Mojsa-Kaja J, Golonka K, Marek T (2015). Job burnout and engagement among teachers: Worklife areas and personality traits as predictors of relationships with work. Int. J. Occup. Med. Environ. Health.

[56] Weißenfels M, Klopp E, Perels F (2021). Changes in teacher burnout and self-efficacy during the COVID-19 pandemic: Interrelations and variables related to change. Front. Educ..

[57] Safta-Zecheria L (2020). Challenges experienced by teachers regarding access to digital instruments, resources, and competences in adapting the educational process to physical distancing measures at the onset of the COVID-19 pandemic in Romania. J. Educ. Sci..

[58] Pletzer JL, Bentvelzen M, Oostrom JK, De Vries RE (2019). A meta-analysis of the relations between personality and workplace deviance: Big Five versus HEXACO. J. Vocat. Behav..

[59] Alarcon G, Eschleman KJ, Bowling NA (2009). Relationships between personality variables and burnout: A meta-analysis. Work Stress..

[60] Hill AP, Curran T (2016). Multidimensional perfectionism and burnout: A meta-analysis. Pers. Soc. Psychol. Rev..

[61] Angelova V, Nasi KH (2020). Burnout syndrome concerning some personality factors among Greek teachers Natasha. AJSS.

[62] Baleghizadeh S, Shayesteh L (2020). Exploring the relationship between teacher burnout, personality traits, and psychological distress among Iranian EFL teachers: A mixed-methods study. J. Lang. Horizons.

[63] Paleksić, V., Ubović, R., Institute of Occupational and Sports Health in Republic of Srpska, Luka, B., Popović, M., & Institute of Occupational and Sports Health in Republic of Srpska,Banja Luka. Personal characteristics and burnout syndrome among teachers of primary and secondary schools. *Sport Med.* **46**, 118–124 (2015).

[64] Ozamiz-Etxebarria N, Idoiaga Mondragon N, Bueno-Notivol J, Pérez-Moreno M, Santabárbara J (2021). Prevalence of anxiety, depression, and stress among teachers during the CoViD-19 pandemic: A rapid systematic review with meta-analysis. Brain Sci..

[65] Tikkanen L, Pyhältö K, Pietarinen J, Soini T (2017). Interrelations between principals’ risk of burnout profiles and proactive self-regulation strategies. Soc. Psychol. Educ..

[66] Hämäläinen R (2021). Understanding teaching professionals’ digital competence: What do PIAAC and TALIS reveal about technology-related skills, attitudes, and knowledge?. Comput. Hum. Behav..

[67] Wang X, Li B (2019). Technostress among university teachers in higher education: A study using multidimensional person-environment misfit theory. Front. Psychol..

[68] Abós S-S, Kim K, García-González (2019). How should stressors be examined in teachers? Answering questions about dimensionality, generalizability and predictive effects using the multicontext stressors scale. IJERPH.

[69] Mérida-López S, Extremera N (2017). Emotional intelligence and teacher burnout: A systematic review. Int. J. Educ. Res..

[70] Răducu C-M, Stănculescu E (2022). Protective factors and teachers’ risk to burnout during the Covid-19 pandemic. Do Kolb’s educator roles matter?—A cluster analysis. IEJEE.

[71] Stănculescu E (2014). Psychological predictors and mediators of subjective well-being in a sample of Romanian teachers. Rev. Cercetare Intervenţ. Soc..

[72] Stănculescu E (2015). Managementul Stresului în Mediul Educațional [Stress Management in Educational Settings].

[73] Iancu AE, Rusu A, Măroiu C, Păcurar R, Maricuțoiu LP (2018). The effectiveness of interventions aimed at reducing teacher burnout: A meta-analysis. Educ. Psychol. Rev..

[74] Meredith C (2020). ‘Burnout contagion’among teachers: A social network approach. J. Occup. Organ. Psychol..

