# Supplementary material for: Personality and socio-demographic variables in teacher burnout during the COVID-19 pandemic: a latent profile analysis
Source: Sci Rep. 2022 Aug 22;12:14272. doi: 10.1038/s41598-022-18581-2 (PMC9395542; doi:10.1038/s41598-022-18581-2)
Supplement: Supplementary file 1 — Supplementary Information. [file 41598_2022_18581_MOESM1_ESM.docx]

Appendix

Descriptive statistics (count and frequency) of sociodemographic variables within five teacher burnout profiles

| Sociodemographic  variables | | First  profile | Second  profile | Third  profile | Fourth  profile | Fifth  profile | Total |
| --- | --- | --- | --- | --- | --- | --- | --- |
| Gender | Female | 175(43.5%) | 87(21.6%) | 27(6.7%) | 62(15.4%) | 51(12.7%) | 402 |
|  | Male | 41(34.2%) | 27(22.5%) | 13(10.8%) | 22(18.3%) | 17(14.2%) | 120 |
| Professional  experience | < 1 year | 27(79.4%) | 4(11.8%) | 0(0.0%) | 0(0.0%) | 3(8.8%) | 34 |
|  | 2 -5 years | 45(64.3%) | 12(17.1%) | 4(5.7%) | 3(4.3%) | 6(8.6%) | 70 |
|  | 5-10 years | 35(40.2%) | 29(33.3%) | 6(6.9%) | 14(16.2%) | 3(3.4%) | 87 |
|  | 10-20 years | 48(35.8%) | 33(24.6%) | 12(9.0%) | 21(15.7%) | 22(14.9%) | 134 |
|  | >20 years | 61(31.0%) | 23(11.6%) | 18(9.1%) | 46(23.4%) | 49(24.9%) | 197 |
| Teaching environment | Urban | 147(43.4%) | 75(22.1%) | 28(8.3%) | 47(13.9%) | 42(12.4%) | 339 |
|  | Rural | 69(37.7%) | 39(21.3%) | 12(6.8%) | 37(20.2%) | 26(14.2%) | 183 |

*Note:* First profile: “No burnout risk”, Second profile: “Low burnout risk”, Third profile: “Cynics”, Fourth profile: “Exhausted and cynics”, Fifth profile: “High burnout risk”.
